# The reliability and validity of the Questionnaire - Children with Difficulties (QCD)

**DOI:** 10.1186/1753-2000-7-11

**Published:** 2013-03-27

**Authors:** Masahide Usami, Daimei Sasayama, Nobuhiro Sugiyama, Nana Hosogane, Soo-Yung Kim, Yushiro Yamashita, Masaki Kodaira, Kyota Watanabe, Yoshitaka Iwadare, Tetsuji Sawa, Kazuhiko Saito

**Affiliations:** 1 Department of Child and Adolescent Psychiatry, National Center for Global Health and Medicine, Kohnodai Hospital, Ichikawa, Japan; 2 Department of Neuropsychiatry, Shinshu University School of Medicine, Matsumoto, Japan; 3 Shibuya Moriya Clinic, Shibuya-Ku, Japan; 4 National Rehabilitation Center for Persons with Disabilities, Tokorozawa, Japan; 5 Department of Pediatrics & Child Health, Kurume University, School of Medicine, Kurume, Japan; 6 Developmental Psychiatry, Graduate School of Medical Science, Kitasato University, Kanagawa, Japan

**Keywords:** Adolescent, Child, Daily life, Questionnaire-Children with Difficulties (QCD), Reliability, Validity

## Abstract

**Background:**

The aim of this study was to evaluate the reliability and validity of the Questionnaire-Children with Difficulties (QCD), which was developed for the evaluation of children’s daily life behaviors during specified periods of the day.

**Methods:**

The subjects were 1,514 Japanese public elementary and junior high school students. For the examination of reliability, Cronbach’s alpha was calculated to assess the internal consistency of the questionnaire. With regard to validity, correlation coefficients were calculated to examine whether QCD scores correlated with those of the ADHD-Rating Scale (ADHD-RS) and the Oppositional Defiant Behavior Inventory (ODBI).

**Results:**

Cronbach’s alpha coefficient for the total score of the QCD was .876. The correlation coefficients of the QCD score with ADHD-RS and ODBI scores were -.514 and -.577, respectively.

**Conclusions:**

The internal consistency and validity of the QCD were demonstrated. The QCD is a reliable and valid instrument for evaluating daily life problems for children during different time periods of the day.

## Background

Children with psychiatric disorders often experience problems in their daily life. Parents also experience difficulties in handling the child’s daily behavioral problems associated with these disorders [1]. In the clinical field, parents’ perceptions of their child’s daily behaviors are believed to be useful in evaluating difficulties associated with mental health disorders in children [1,2]. The Child Behavior Checklist (CBCL) [3], the Strength and Difficulties Questionnaire (SDQ) [2,4] and the Weiss Functional Impairment Rating Scale (WFIRS) [3,5] are commonly used parent/caregiver questionnaires in the field of child and adolescent psychiatry for the evaluation of a child’s functioning in daily life. The Japanese version of CBCL and SDQ were introduced in Japan in 2003 and 2008 [3,4], respectively, and have been widely used in Japan. CBCL covers more psychopathological dimensions. However, there are several problems with these questionnaires. Due to the large number of parameters, the CBCL and WFIRS are not convenient to use in daily practice. Furthermore, the CBCL, SDQ, and WFIRS were not developed to evaluate the child’s behavior during each period of the day. Furthermore, there is no Japanese version of the WFIRS.

Attention deficit/hyperactivity disorder (ADHD) and oppositional defiant disorder (ODD) are common disorders among children and adolescents [5,6]. Children with ADHD and/or ODD usually have many difficulties at various times of the day and during numerous activities such as homework, family events, and playing with friends [6-12]. Nevertheless, comprehensive treatment consisting of parent training based on learning principles and behavioral therapy, psychotherapy, social therapy, and medication is recommended in the guidelines for the treatment of ADHD in Japan [13]. Medication is usually attempted after other behavioral approaches, such as parent training and psychotherapy. However, only two drugs, long-acting methylphenidate and the non-stimulant atomoxetine, are currently approved for the treatment of ADHD in Japan [13]. Both stimulants and atomoxetine are recommended as options for the management of ADHD and/or ODD in children and adolescents. The decision regarding which product to use is a very important issue in clinical settings [14], and partly depends on whether a continuous effect of the medication throughout the day is required. The ADHD-Rating Scale (ADHD-RS) and Oppositional Defiant Behavior Inventory (ODBI) have been most widely used to evaluate hyperactivity, impulsiveness, inattention, and oppositional defiant behaviors of children [4,15-20]. However, the ADHD-RS and ODBI only target these symptoms and do not include an assessment of the difficulties of daily life. Furthermore, these questionnaires do not inquire about behaviors during specific periods of the day.

In Japan, the Questionnaire-Children with Difficulties (QCD), constructed by Yamashita, has been widely used in order to evaluate parents’ perceptions of their child’s daily behaviors during specific periods of the day such as morning, school, after school, evening, and night time [5,14]. The QCD has three important characteristics: the capability of evaluating life function, the capability of performing an all-day-long evaluation, and the convenience of use in daily practice [3,14]. One of the major advantages of the QCD is that it includes only 20 questions and is convenient to use. Only a short period of time is required to fill out the QCD, compared with the CBCL and WFIRS. The QCD is also practical for sharing information among caretakers, because it enables the evaluation of life function at each period of the day [6,14]. The ADHD-RS, ODBI, and SDQ can also be easily administered because of the small number of questions. However, these do not evaluate activities during specific periods of the day. In this respect, the QCD is superior in clarifying problems encountered by children with ADHD and/or ODD throughout the day. It exhibits high user-friendliness in clinical practice. The use of the QCD in ADHD and/or ODD children enables one to elucidate problems in their daily life during different time periods of the day; it is also expected to provide clinicians with necessary information for selecting appropriate drug therapy.

With this background, the QCD has been developed for use in evaluating children with ADHD. However, the reliability and validity of the QCD has not been evaluated and there have been no studies using the QCD. Thus, in order to use the QCD in future clinical studies, it is critical to evaluate the internal consistency, convergent validity, and concurrent validity of the QCD in Japanese elementary school and junior high school students.

A major hypothesis of the study was that a satisfactory internal consistency would be found separately for the total score and subscores of the QCD, and that the convergent and concurrent validity of the QCD would also be elucidated separately for the total score and subscores of the QCD. This hypothesis predicted that the total score and subscores of the QCD would exhibit significantly high correlations with ADHD-RS and ODBI scores. We found that QCD scores associated with the daily difficulties of children with ADHD and ODD symptoms.

## Subjects and methods

### Study design and setting

This was a survey study using a paper questionnaire. The Kohnodai Hospital is located in Ichikawa City, and its primary service area is Chiba Prefecture, including Ichikawa City and the eastern part of the Tokyo metropolitan area. Unfortunately, we have no valid data about the socioeconomic status of Ichikawa City citizens. Ichikawa City is situated in the western part of Chiba Prefecture, facing Tokyo, across the Edogawa River. Located approximately 20 kilometers away from the Tokyo metropolitan area, the city has fully developed into a residential area and center for education. The population is estimated at 471,104 (as of April 2008), making it the fourth largest city in the prefecture.

### Recruitment and participants

This survey was conducted as part of the school education program under the initiative of the Board of Education in Ichikawa City. The QCD, ADHD-RS, and ODBI were distributed by teachers to the parents of 10,242 randomly selected children at 11 public elementary schools (7111 children) and 5 junior high schools (3131 children) in Ichikawa City, Chiba Prefecture. The survey was carried out in July 2010.

First, the survey method was explained to the principals of all of the schools by the Education Committee of Ichikawa City. Subsequently, teachers distributed a letter explaining the survey, which had been constructed by the Education Committee, to all children and their parents. The letter clearly stated that the parent filling out the consent form would be considered (for both the parents and the students) as having given consent to the survey. The letter also specified that the survey results would be used to provide children with psychological care to facilitate their education at school and that the results would be published as a medical paper.

The parents were asked to fill out all three questionnaires. Questionnaires were retrieved from 1,803 parents who gave informed consent to the mailed survey. Of them, 1,514 questionnaire sheets (84%) that were filled out completely were analyzed. We excluded 289 questionnaire sheets (16%) that were filled out incompletely.

The 1,514 subjects comprised 752 boys and 762 girls. The average age was 9.7 ± 2.5 (mean ± S.D.; range, 6–15) years. The average ADHD-RS score was 9.6 ± 15.5 points. The average ODBI score was 13.0 ± 9.9 points, and 235 children (15.5%) had scores exceeding the cut-off value of 24 [20].

This study was approved by the ethical committee of the National Center for Global Health and Medicine.

### Measures

#### Questionnaire-Children with Difficulties (QCD)

The QCD comprises 20 questions related to activities that occur during specific periods of the day: Questions No. 1–4, early morning/before going to school; No. 5–7, school; No. 8–10, after school; No. 11–14, evening; No. 15–18, night; and No. 19 and 20, overall behavior (Additional file 1). Each question is scored in four grades: 0 = completely disagree, 1 = somewhat (partially) agree, 2 = mostly agree, and 3 = completely agree. Higher scores indicate higher life functioning and less difficulty in children’s daily activities that occur during specific periods of the day. The questionnaire is composed of practical and easy-to-understand questions inquiring about basic daily activities, such as washing one’s face, brushing one’s teeth, and getting dressed. These subscales of the QCD were composed according to the clinical experiences of Yamashita in order to match the daily life of Japanese children without using factor analysis [5,14]. The appropriate targeted age range of children (whose parents answer the QCD) was the elementary school and junior high school student.

#### ADHD-rating scale (ADHD-RS)

The ADHD-RS comprises 18 questions about hyperactivities, impulsiveness, and inattention. Four possible responses are recorded to each question, ‘Generally none,’ ‘Usually none,’ ‘Usually exists,’ or ‘Always exists,’ that were scored as 0, 1, 2, or 3 points, respectively, and the total points were used for evaluation. Higher scores indicate greater numbers of symptoms and severe symptoms. ADHD-RS is widely used in the Japanese clinical field and in clinical research.

#### Oppositional Defiant Behavior Inventory (ODBI)

The Oppositional Defiant Behavior Inventory (ODBI) was developed and standardized by Harada and colleagues [20]. The ODBI comprises 18 items to be answered by the caregiver. Four possible responses are recorded to each question, ‘Generally none,’ ‘Usually none,’ ‘Usually exists,’ or ‘Always exists,’ that were scored as 0, 1, 2, or 3 points, respectively, and the total points were used for evaluation. The cut-off point of ODBI was 20 points and subjects whose ODBI score was over 20 points were considered to be in the high ODBI subgroup. The higher the score, the greater the severity of oppositional defiant behavior the subjects exhibited.

### Statistical analyses

#### Distribution of the QCD scores

Scores on the 20 questions of the QCD, scores on questions in each of the 6 subcategories, and the total score were determined separately, and the percentage of parents who gave a particular answer to each question, the average score, and the standard deviation were calculated. The normal distribution of QCD scores was examined using the Kolmogorov-Smirnov test.

#### Reliability and validity of the QCD

The reliability and validity of the QCD were examined with the scores for the 20 questions in the QCD. For the examination of reliability, Cronbach’s alpha, a marker of internal consistency, was calculated separately for the total score and subscores in the case of a normally distributed QCD score. If QCD scores were not normally distributed, we calculated the McDonald’s omega. Correlations across the full scale of 20 items were calculated.

With regard to convergent validity, Pearson’s correlation coefficient was calculated to examine whether the total score and subscores of the QCD correlated with the hyperactivity and inattention scores on the ADHD-RS and the ODBI. Although there is no cut-off value in the ADHD-RS, based on the fact that there is a cutoff value in the ODBI, concurrent validity was analyzed using the ODBI score. The effects of gender (males, females) and ODBI score group (high score group, low score group) on the total QCD score were analyzed using two-way analysis of variance. The high ODBI score group included those with ODBI score of 24 or greater, and the low score group included those with ODBI score of less than 24.

All statistical tests were two-tailed, and *P* < 0.05 indicated statistical significance. Analyses were performed using PASW Statistic 18.0 (IBM Japan Inc.).

## Results

### Distribution of the QCD scores

QCD surveys that were filled out completely were collected from the parents of 1,514 children. All Kolmogorov-Smirnov test results were significant at p < 0.001. The total score and subscores of the QCD were normally distributed.

The mean of the total score of the QCD was 13.0 ± 9.9 (mean ± S.D.). The percentage of respondents who gave each answer for each question and the mean for each question are shown in Table 1. All questions except for the first two had mean scores of 2 or higher.

**Table 1:** QCD scores among the parents of 1514 elementary school and junior high school students

	**Answer**							
	**0=completely disagree**	**1=somewhat (partially) agree**	**2=mostly agree**	**3=completely agree**	**N**	**Mean**	**SD**	**Correlations coefficients with the full scale**
Early morning/before going to school								
1. Can your child promptly get out of his/her bed?	213(14%)	381(25%)	482(32%)	438(29%)	1514	1.76	1.02	.454
2. Can your child promptly groom himself/herself (for example, washing face, brushing teeth and getting dressed)?	131(9%)	394(26%)	528(35%)	461(30%)	1514	1.87	0.95	.586
3. Can your child behave in an age-appropriate manner at breakfast?	30(2%)	238(16%)	559(37%)	687(45%)	1514	2.26	0.79	.617
4. Can your child spend his/her time before going to school in the morning without getting into trouble or having quarrels with his/her parents or siblings?	53(4%)	259(17%)	549(36%)	653(43%)	1514	2.19	0.84	.613
School								
5. Does your child like going to school?	19(1%)	113(7%)	434(29%)	948(63%)	1514	2.53	0.69	.500
6. Can your child behave in class as other children do?	16(1%)	93(6%)	399(26%)	1006(66%)	1514	2.58	0.66	.673
7. Does your child have friends who accept him/her at school?	8(1%)	72(5%)	393(26%)	1041(69%)	1514	2.63	0.60	.612
After school								
8. Can your child discuss events that happened at school with his/her parents/guardian?	25(2%)	230(15%)	545(36%)	714(47%)	1514	2.29	0.78	.636
9. Does your child have friends of his/her own age?	5(1%)	60(4%)	294(19%)	1155(76%)	1514	2.72	0.55	.621
10. Can your child confidently participate in extracurricular activities, such as sports, with children of his/her own age?	48(3%)	165(11%)	365(24%)	936(62%)	1514	2.45	0.81	542
Evening								
11. Can your child do his/her homework at home without difficulties?	41(3%)	207(14%)	450(30%)	816(54%)	1514	2.35	0.81	.664
12. After everyone returns home (including parents/guardians), can your child enjoy family time without constantly quarrelling with others?	48(3%)	256(17%)	614(41%)	596(39%)	1514	2.16	0.81	.662
13. Can your child converse in a calm manner during dinnertime conversations?	12(1%)	130(9%)	506(33%)	866(57%)	1514	2.47	0.68	.675
14. Do parents feel comfortable being together with the child when engaging in activities (for example, going out or shopping)?	18(1%)	80(5%)	334(22%)	1082(71%)	1514	2.64	0.64	.599
Night								
15. Adolescent child (12 years or older):								
Can your child engage in activities at night with friends of his/her own age? These activities may include playing, studying, going to cram school, taking private lessons (for example, playing a musical instrument and/or calligraphy), and playing sports.	13(3%)	33(8%)	94(22%)	290(67%)	430	2.54	0.76	.533
16. Younger children (younger than 12 years):								
Can your child follow instructions at night (for example, brushing teeth, changing clothes)?	9(1%)	89(8%)	317(21%)	667(44%)	1082	2.52	0.68	.616
17. Can your child go to sleep without any difficulties?	11(1%)	76(5%)	261(17%)	1166(77%)	1514	2.71	0.59	.567
18. Is your child sleeping without waking up during the night?	9(1%)	50(3%)	263(17%)	1192(79%)	1514	2.74	0.54	.443
Overall behavior								
19. Does your child have self-confidence? Is your child socially accepted by others (such as belonging to a group of his/her friends), and emotionally stable?	24(2%)	129(9%)	556(37%)	805(53%)	1514	2.41	0.71	.747
20. Does your child have more days in the week, where he/she is able to spend the day without facing confusion, getting into quarrels or displaying rebellious behavior?	46(3%)	182(12%)	550(36%)	736(49%)	1514	2.31	0.80	.696

All correlations were significant at p < 0.001.

### Reliability and validity of the QCD

With regard to the reliability of the QCD, Cronbach’s alpha for the subscores ranged from .569 to .775, and the alpha for the total score was .876 (Table 2). Correlation coefficients of each question’s scores with total QCD scores were .443 and .747, respectively (Table 1).

**Table 2:** Internal consistency and convergent validity of the QCD

**Subscore of QCD**	**Cronbach’s α**				
		**ODBI**	**ADHD-RS**		
			**Hyperactivity**	**Inattention**	**Total score**
Early morning/before going to school	0.756	−0.419	−0.332	−0.448	−0.440
School	0.736	−0.241	−0.257	−0.355	−0.348
After school	0.680	−0.256	−0.279	−0.411	−0.398
Evening	0.775	−0.552	−0.429	−0.542	−0.547
Night	0.569	−0.262	−0.243	−0.279	−0.291
Overall behavior	0.728	−0.505	−0.400	−0.457	−0.474
Total	0.876	−0.514	−0.448	−0.575	−0.577

All correlations were significant at p < 0.001.

As to the convergent validity of the QCD, the correlation coefficients between the OBDI score and the QCD subscores ranged from -.241 to -.552. The correlation coefficients between the ADHD-RS score and the QCD subscores ranged from -.243 to -.577. All correlations were significant at p < 0.001. Low correlations were observed between ‘night’ and the hyperactivity/impulsiveness score, the attention deficit score, and the total ADHD-RS score, and the ODBI score. With regard to concurrent validity, the QCD score of the low ODBI score group was significantly higher than that of the high ODBI score group (Table 3, F (1,1369) = 5.31, p < 0.01).

**Table 3:** Scores of the QCD by gender and ODBI score group (Low score group vs. High score group)

	**Male**			**Female**						
	**Mean**	**SD**	**N**	**Mean**	**SD**	**N**		**F**	**df**	**p**
							Gender × ODBI score group	0.00	1,1510	NS
Low score group (ODBI<23)	50.19	21.48	632	54.77	72.52	647	Gender	1.64	1,1510	NS
High score group (ODBI≧23)	40.20	13.42	120	44.66	25.63	115	ODBI score group	8.00	1,1510	0.0047

The QCD total score showed a significant correlation with the ODBI score and the hyperactivity/impulsiveness score, attention deficit score, and total score of the ADHD-RS.

## Discussion

### Distribution of the QCD

In the present study, 1,514 Japanese public elementary and junior high school students were examined to confirm the internal consistency and validity of the QCD.

QCD scores were normally distributed. Table 2 provides useful table for the clinical field and clinical studies to compare any child/adolescent patient with these data.

### Reliability of the QCD

The internal consistencies for both the total score and for the subscores of the QCD were found to be satisfactory. However, in future study, retests must be administered to measure test-retest reliability.

### Validity of the QCD

The convergent validity was found to be satisfactory for both the total score and subscores of the QCD. We found that the total score of the QCD was significantly correlated with scores on the ADHD-RS and ODBI, which assess the severity of ADHD symptoms and oppositional defiant behaviors, respectively. However, subscores of the QCD exhibited a broad range of correlations (−.243 to -.577) with scores on the ADHD-RS and ODBI. The ‘night’ score displayed a low correlation with ADHD-RS and ODBI scores. As ADHD and/or ODBI symptoms were not associated with the ‘night’ score, the ‘night’ score may be associated with other problems in children. This study did not evaluate sleep problems/disorders in the subjects. Therefore, it is impossible to explain these low correlations with data from this study alone. In order to better explain these results, it will be necessary to perform clinical studies for children with ADHD using the QCD.

The concurrent validity was found to be satisfactory for both the total score and subscores of the QCD and the score of the ODBI. These results suggest that the total QCD score can be used to evaluate the daily life of children with ODD symptoms in Japan.

The validity of the QCD was examined with attention deficit, hyperactivity/impulsiveness, and oppositional defiant behaviors, which are all major aspects of child-care difficulties for parents. However, further comparison with well-established scales evaluating children’s’ daily life functions, such as the CBCL, would be necessary to further confirm the validity of the QCD on assessing daily functions.

### Using the QCD

This study revealed that a child’s functioning in daily life over an entire day as measured by the QCD was related to the ADHD-RS score and the ODBI score. Thus, the present findings suggest that the QCD possesses sufficient validity to assess daily life difficulties caused by hyperactivity, inattention, and oppositional behaviors. Only the ‘night’ subscale did not indicate the difficulties caused by hyperactivity, inattention, and oppositional behaviors. In the case of a high ‘night’ subscore, the clinician should pay attention to the presence of comorbid disorders, such as sleep disorder.

This study has some limitations that need to be considered. The subjects were recruited from the general population in one district in Japan. The response rate of about 15 % has to be discussed. Therefore, the present findings cannot be generalized to clinical populations. Furthermore, a district-specific bias may have influenced the results. For example, the regional school schedule may have predisposed hyperactive children to have difficulties during certain periods of the day. Such factors must be taken into account when determining the reference values and the cut-off scores. Future studies should include participants from different districts to minimize the effects of district-specific factors. However, the large sample size in the present study allowed us to draw newly valuable conclusions.

## Conclusion

The internal consistency and convergent validity were found to be satisfactory for both the total score and subscores of the QCD. The concurrent validity of the QCD was elucidated separately for the total QCD score. Use of the QCD in children enables one to conveniently evaluate the problems in their daily life during specific time periods of the day.

## Additional file

## Competing interests

The authors declare that they have no competing interests.

## Authors’ contributions

MU was the corresponding author. MU was responsible for the data collection, and for writing the manuscript. MU planned and supervised the research project. MK, KW, YI, and KS were responsible for the data collection, and for writing the manuscript. ST, DS, and NS was responsible for the statistical analyses, and took part in writing the manuscript. DS, NS, NH, SK, and YY took part in writing the Additional file 1. All authors took part in reviewing draft versions of the manuscript, and approved of the final version.

## Supplementary Material

Additional file 1 Questionnaire-Children with Difficulties (English version*). Click here for file
